# Myocarditis Following COVID-19 BNT162b2 Vaccination Among Adolescents in Hong Kong

**DOI:** 10.1001/jamapediatrics.2022.0101

**Published:** 2022-02-25

**Authors:** Xue Li, Francisco Tsz Tsun Lai, Gilbert T. Chua, Mike Yat Wah Kwan, Yu Lung Lau, Patrick Ip, Ian Chi Kei Wong

**Affiliations:** 1 Department of Medicine, School of Clinical Medicine, Li Ka Shing Faculty of Medicine, The University of Hong Kong, Hong Kong; 2 Centre for Safe Medication Practice and Research, Department of Pharmacology and Pharmacy, Li Ka Shing Faculty of Medicine, The University of Hong Kong, Hong Kong; 3 Department of Paediatrics and Adolescent Medicine, School of Clinical Medicine, Li Ka Shing Faculty of Medicine, The University of Hong Kong, Hong Kong; 4 Department of Paediatrics and Adolescent Medicine, Princess Margaret Hospital, Hong Kong

## Abstract

This cohort study assesses the association between the single-dose COVID-19 BNT162b2 vaccination regimen and myocarditis risk among vaccinated adolescents in Hong Kong before and after the single-dose policy.

Cases of myocarditis following the second dose of messenger RNA (mRNA) vaccine are accruing worldwide, especially in younger male adults and adolescents.^1,2,3,4^ In weighing the risk of myocarditis against the benefit of preventing severe COVID-19, Norway, the UK, and Taiwan have suspended the second dose of mRNA vaccine for adolescents. Similarly, adolescents (aged 12-17 years) in Hong Kong have been recommended to receive 1 dose of BNT162b2 instead of 2 doses 21 days apart since September 15, 2021 (Figure).

**Figure.**
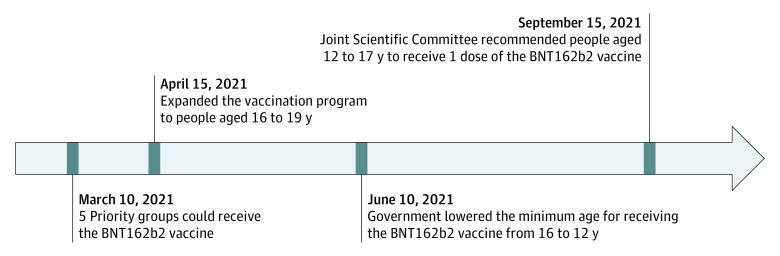
Timeline of BNT162b2 Vaccination Policy Among Adolescents in Hong Kong

## Methods

This cohort study was conducted before the arrival of the Omicron variant. We linked vaccination records with the Hong Kong territorywide electronic health record database through government-commissioned population-based COVID-19 vaccine safety surveillance.^3^ Among adolescents who received at least 1 dose of BNT162b2 between March 10 and October 18, 2021, inpatient myocarditis cases were identified using the *International Classification of Diseases, Ninth Revision, Clinical Modification* (422.x and 429.0). Adolescents with a history of myocarditis were excluded. The study was approved by the institutional review board of the University of Hong Kong/Hospital Authority Hong Kong West Cluster and the Department of Health Ethics Committee with a waiver of informed consent because anonymized data were used. The statistical tests are described in the eMethods in the Supplement. This study followed the Strengthening the Reporting of Observational Studies in Epidemiology (STROBE) reporting guideline.

## Results

A total of 224 560 first doses and 162 518 second doses of BNT162b2 were administered to adolescents. Forty-three adolescents had myocarditis-related hospitalization following receipt of BNT162b2 vaccination, and 84% of the hospitalizations (36 of 43) occurred after the second dose. The incidence rate was 3.12 (95% CI, 1.25-6.42) and 22.15 (95% CI, 15.51-30.67) per 100 000 persons for the first and second dose, respectively (Table). The number needed to harm for the first and second dose were 32 051 and 4515, respectively. The crude risk ratio of the second dose vs first dose was 7.11 (95% CI, 3.16-15.97). The cumulative incidence of myocarditis decreased from 43 cases in 202 315 adolescents vaccinated (21.25, 95% CI, 15.38-28.63) per 100 000 persons to 0 cases in 22 245 adolescents vaccinated at implementation of the single-dose policy. The 40 167 prepolicy first dose recipients did not receive the second dose because of the single-dose policy. Based on the number needed to harm of the second dose, an estimated 8.90 (95% CI, 6.23-12.32) myocarditis cases were prevented.

**Table.** Myocarditis Cases Following the BNT162b2 Vaccination Among 43 Adolescents in Hong Kong Before and After the Single-Dose Policy

Variable	Male (n = 38 [88%])	Female (n = 5 [12%])	Total (n = 43 [100%])
Age, mean (SD), y	14.95 (1.35)	14.20 (2.17)	14.86 (1.46)
Before single-dose recommendation			
Cases, No.	38	5	43
Doses administered, total No.	181 392	177 405	358 797
Adolescents who received vaccination, total No.	102 242	100 073	202 315
After single-dose recommendation			
Cases, No.	0	0	0
Doses administered, total No.	14 386	13 895	28 281
Adolescents who received vaccination, total No.	11 525	10 720	22 245
Overall observational period			
Cases after first dose, No./recipients of first dose, total No.	6/113 767	1/110 793	7/224 560
Incidence after first dose per 100 000 persons (95% CI)	5.27 (1.94-11.48)	0.90 (0.023-5.03)	3.12 (1.25-6.42)
NNH for the first dose (95% CI)	18 975 (8711-51 546)	111 111 (19 881-4 347 826)	32 051 (15 576-80 000)
Cases after second dose, No./recipients of second dose, total No.	32/82 011	4/80 507	36/162 518
Incidence after second dose per 100 000 persons (95% CI)	39.02 (26.69-55.08)	4.97 (1.35-12.72)	22.15 (15.51-30.67)
NNH for the second dose (95% CI)	2563 (1816-3747)	20 121 (7862-74 074)	4515 (3261-6447)

Abbreviation: NNH, number needed to harm.

## Discussion

In this cohort study, the single-dose regimen was found to be associated with reduction in myocarditis risk among vaccinated adolescents. Limitations include sample size during the postpolicy period. Since May 2021, no local transmission of SARS-CoV-2 has occurred in Hong Kong, with stringent nonpharmaceutical interventions. Among the 343 700 adolescents in Hong Kong, no COVID-19-related death has been reported, and the only one admitted to the pediatric intensive care unit due to COVID-19 was an imported case,^5^ indicating that the risk of death or complications from COVID-19 is extremely low among adolescents in Hong Kong. Vaccination policy for adolescents should consider the trade-off between risks and benefits. In countries with large outbreaks and to prevalent local transmission, the risk-benefit assessment would favor a 2-dose regimen because the single-dose regimen provides suboptimal protection from severe outcomes associated with COVID-19. However, in settings with no evident local transmission and stringent infection control policies, single-dose mRNA vaccination might be a viable option for offering protection to adolescents from severe outcomes associated with COVID-19.

Nevertheless, questions remain about the mechanism of myocarditis following mRNA vaccine. Potential ways to reduce myocarditis risk in adolescents could be the use of single-dose only, a lower dosage for 2 doses as recommended for children aged 5 to 11 years,^6^ or a lengthened interval between doses. More laboratory, trial, and postmarketing data may become available to answer these questions. Our study expands the current understanding of dose-response relationship and suggests that COVID-19 vaccination recommendations in adolescents may need to be customized rather than standardized to fit all.

## Notes

Supplement. **eMethods.** Statistical Methods
