# Supplementary material for: Myocarditis Following COVID-19 BNT162b2 Vaccination Among Adolescents in Hong Kong
Source: JAMA Pediatr. 2022 Feb 25;176(6):612–4. doi: 10.1001/jamapediatrics.2022.0101 (PMC9171561; doi:10.1001/jamapediatrics.2022.0101)
Supplement: Supplement. — eMethods. Statistical Methods [file jamapediatr-e220101-s001.pdf]

Supplemental Online Content

Li X, Lai FTT, Chua GT, et al. Myocarditis Following COVID-19 BNT162b2 Vaccination Among Adolescents in Hong Kong. *JAMA Pediatr*. Published online February 25, 2022. doi:10.1001/jamapediatrics.2022.0101

eMethods. Statistical Methods

This supplemental material has been provided by the authors to give readers additional information about their work.

© 2022 Li X et al. *JAMA Pediatrics.*

eMethods. Statistical Methods

Descriptive statistics were applied to summarize the characteristics of myocarditis cases following the first or second doses of COVID-19 vaccination. Cumulative incidence with exact 95% confidence interval (CI) were estimated based on Poisson distribution. The crude risk ratio and 95% CI of myocarditis between the second and the first dose were estimated using riskratio() function from R package epitools. Fisher's exact test was used to examine the impact of single-dose policy by comparing the cumulative incidence before and after the policy implementation. Number needed to harm (NNH) following the second and first dose were calculated based on the corresponding incidence estimates of myocarditis. Results were reported among the total population and in subgroups by sex. R version 4.0.3 was used for all statistical tests. Two-side P values of less than 0.05 were considered significant. All results were independently cross-checked by at least two investigators for quality assurance.

© 2022 Li X et al. *JAMA Pediatrics.*
